# New Imaging Techniques on the Horizon to Study Overactive and Neurogenic Bladder

**DOI:** 10.1007/s11884-025-00775-9

**Published:** 2025-03-05

**Authors:** Nyasia M. Jones, Ethan S. Casto, Linda S. Burkett, John E. Speich, Alejandro Roldán-Alzate, Adam P. Klausner

**Affiliations:** 1 Department of Surgery, Division of Urology, Virginia Commonwealth University School of Medicine, PO Box 980118, Richmond, VA 23298-0118 USA; 2 Department of Obstetrics and Gynecology, Virginia Commonwealth University School of Medicine, Richmond, VA USA; 3 Department of Mechanical & Nuclear Engineering, Virginia Commonwealth University College of Engineering, Richmond, VA USA; 4 Departments of Mechanical Engineering and Radiology, University of Wisconsin, Madison, WI USA

**Keywords:** Overactive bladder, Neurogenic bladder, Imaging, Urodynamics

## Abstract

**Purpose of Review:**

This review will focus on the current usage and the potential future applications of new imaging techniques on the horizon to study overactive and neurogenic bladder.

**Recent Findings:**

Bladder Near-Infrared Spectroscopy (NIRS) has been used to non-invasively identify bladder outlet obstruction, detrusor overactivity, and other forms of voiding dysfunction, but motion artifact has been a limiting factor preventing widespread adaptation. However, newer NIRS units employ accelerometers which enable isolation and splicing of motion and on-going studies show renewed promise for bladder NIRS. Ultrasound has been successfully used to evaluate bladder outlet obstruction and other forms of LUT. Techniques including m-mode evaluation of micromotion, as well as the assessment of bladder wall thickness, bladder weight, shape/circularity, vibrometry, and elastography have been explored. Dynamic bladder functional magnetic resonance imaging (fMRI) is the newest bladder imaging technology on the horizon and provides a novel method to assess bladder function alongside real-time high-resolution 3D anatomic images.

**Summary:**

Bladder imaging techniques including NIRS, ultrasound, and functional fMRI have been developed and are now being used as noninvasive techniques that could potentially supplement, or even replace, traditional Urodynamics.

## Introduction

### Limitations of Current Multichannel Urodynamics Testing

Multichannel urodynamic studies (UDS) are currently the gold standard for assessing lower urinary tract dysfunction (LUTD) and determining a cause for lower urinary tract symptoms (LUTS) [1–3]. LUTD include alterations of voiding and storage, such as urinary incontinence, urinary retention, overactive bladder (OAB), and neurogenic urinary tract dysfunction. The goal of UDS is to identify the underlying causes of LUTD, which may include bladder outlet obstruction, poor bladder compliance, involuntary contractions of the detrusor muscle, and/or a poorly contractile detrusor [1, 3].

However, there are many limitations associated with UDS which may affect the quality and repeatability of the results. These include the requirement of invasive catheters placed in the urethra and vagina/rectum, and bladder fill rates that are typically supra-physiologic. UDS findings are poorly reproducible, especially for the detection of detrusor overactivity. One study found UDS to be poorly reproducible both between consecutive fills during the same visit and across repeated study visits. This could be attributed to physiological fluctuations in bladder function and/or a lack of sensitivity in the UDS instruments [4]. Another study demonstrated that UDS patients report increases in sensation, even with sham filling, highlighting the potential effects of catheterization and acquiescence bias [5]. Furthermore, a recent study found that maximum cystometric capacity correlated poorly with maximum voided volumes reported on bladder diaries by women with OAB [6]. Other studies have identified high rates of artifacts in UDS studies performed on healthy subjects without any LUTS [7, 8]. Additionally, patients often report embarrassment and discomfort during UDS. To further complicate matters, UDS testing is expensive and has been associated with complications that include bleeding, pain and urinary tract infections [9].

There is a clear need to improve UDS diagnostics, and a range of refinements and new technologies have been explored [10, 11]. This review will focus on the current usage and the potential future applications of new imaging techniques on the horizon to study OAB and neurogenic bladder. In this regard, bladder imaging techniques including near-infrared spectroscopy (NIRS), ultrasound, and functional magnetic resonance imaging (fMRI) will be reviewed. These tools allow for noninvasive and cost-effective imaging that could hopefully one day supplement, or even replace, traditional UDS.

### Bladder Near-Infrared Spectroscopy (NIRS)

#### What is NIRS?

NIRS utilizes a photodiode to detect near infrared light reflected through tissue to quantify the relative concentrations of oxygenated and deoxygenated hemoglobin [12]. While most often used for the imaging of brain neuroexcitation and cerebral blood flow [13], NIRS has been expanded to applications in urology [12]. Koven and Herschorn reviewed applications of NIRS in urology in 2022 [14], and Tu et al., reviewed the use of NIRS for bladder volume measurement in 2023 [15]. The present review will briefly cover NIRS applications for bladder imaging, specifically in cases of bladder outlet obstruction (BOO), OAB, underactive (UAB), painful bladder, and in functional pediatric urology conditions.

Stothers and colleagues defined NIRS patterns throughout the micturition cycle in asymptomatic children and adults [16]. They monitored oxygenated, deoxygenated, and total hemoglobin and identified reproducible trends where total and oxygenated hemoglobin increased after participants were given permission to void, but prior to the onset of voiding, and then slowly returned to baseline values following the completion of voiding. This study demonstrated that changes in bladder hemodynamics could be quantified throughout voiding and highlighted the potential use of NIRS as a diagnostic technique for LUTD [16].

## Use of Bladder NIRS to Assess LUTD

Bladder NIRS has been investigated as a tool to diagnose non-neurogenic OAB, “a complex syndrome characterized by urinary urgency, usually with frequency and nocturia, with or without urgency urinary incontinence, in the absence of urinary tract infection or other pathology [17].” NIRS has been used to study bladder hemodynamics in individuals with detrusor overactivity (DO) identified via UDS, with the goal of identifying distinctive NIRS patterns. Several investigators have reported statistically significant increases in deoxyhemoglobin associated with DO which was not observed during voluntary voiding. The change in the NIRS during DO was strongly associated with DO with AUC values of 0.80–0.85 for hemoglobin and 0.73–0.84 for deoxyhemoglobin curves [18, 19].

As expected, there are questions regarding the reliability and reproducibility of NIRS techniques for the diagnosis of DO. Mastoroudes et al. evaluated the sensitivity and specificity of bladder NIRS when compared to UDS for the detection DO in women with OAB. NIRS was demonstrated to have a sensitivity of 80.6%, but a specificity of only 28.1%. This ultimately led the team to dismiss NIRS as unreliable in detecting DO [20]. However, these findings could have been related to the relatively high body mass index (BMI) or darker skin pigmentation of individuals in the study, and/or the limited availability of techniques to separate true NIRS signals from background noise and account for artifacts due to participant motion. As a result, the investigators felt, at that time, that NIRS did not have enough fidelity to be used as an effective diagnostic tool for the detection of DO [14]. A study by Stothers et al., suggested multiple distinctive NIRS patterns for individuals with OAB based on its multicausal and unknown mechanisms [16]. Furthermore, their work suggests that the NIRS criteria used to identify DO in the Mastoroudes study [20] may have lacked the sophistication and controls necessary to identify all of the potential variations of DO [20].

In contrast to OAB, UAB is characterized by a “slow urinary system, hesitancy and straining to void, with or without a feeling of incomplete bladder emptying, sometimes with storage symptoms [21].” Detrusor underactivity (DU) is a urodynamic finding associated with UAB, and DU is defined as “a contraction of reduced strength and/or duration, resulting in prolonged bladder emptying and/or a failure to achieve complete bladder emptying within a normal time span [21].” Clinically, distinguishing between UAB and BOO can be difficult in men with LUTS. Although some investigators presented promising preliminary data showing that bladder NIRS could potentially identify detrusor underactivity during UDS, no confirmatory full-length papers have reported using this or alternative methodologies [22].

The most well studied association of NIRS with LUTD has been in individuals with BOO. Macnab and Stothers compared NIRS tracings between individuals with BOO and healthy controls, and found a decrease in total and oxyhemoglobin during the voiding phase within the BOO cohort [23]. This result supported their hypothesis that the oxygen demand exceeds the supply in obstructed voiding, which is a trend also observed following ischemia and hypertrophy in the heart due to increased pressure from hypertension or outflow obstruction [24]. These and other findings have led to the development of NIRS algorithms aimed at noninvasive diagnosis of BOO with comparative accuracy to invasive UDS [16]. One study combined NIRS parameters with the maximum flowrate measured during uroflowmetry and the post-void residual volume to identify BOO with a sensitivity of 87.7% and specificity of 88.9%. A later version of this algorithm was found to have a specificity and precision of 88% and 94%, respectively [16, 25].

Painful bladder is defined as “an unpleasant sensation perceived to be related to the urinary bladder, associated with LUTS of more than 6-week duration, in the absence of infection or other identifiable causes [26].” The pathogenesis of painful bladder remains uncertain and is most likely multifactorial, and evidence gathered from bladder biopsies in patient populations with interstitial cystitis/bladder pain syndrome (ICS/BPS) implicates chronic submucosal inflammation [27]. Shadgan et al. postulated that inflammation may lead to changes in tissue oxygenation parameters that could potentially be quantified using bladder NIRS for the diagnosis of IC/BPS. They tested four patients with IC/BPS and 20 with LUTD without IC/BPS, and found that participants with IC/BPS exhibited a significantly larger detrusor tissue saturation index, as a measure of tissue oxygenation [28].

The work-up and evaluation of children with LUTD using UDS is challenging because of the need for invasive urethral and rectal catheterization. In this regard, non-invasive technologies such as NIRS may represent extremely useful tools for future investigation [14]. Previous studies evaluated bladder NIRS in children with LUTD (3–14 years old) along with asymptomatic controls. NIRS parameters were recorded from permission to void until the completion of voiding. The investigators found that controls displayed similar NIRS patterns as asymptomatic adults (increased total hemoglobin after participants received permission to void and during urination). However, the children with LUTD displayed reduced or no increase in total hemoglobin throughout micturition [29]. These results imply insufficient hemodynamic priming for an effective bladder contraction and the possibility of muscle fatigue during voiding, a similar finding to adult males with BOO.

## Looking to the Future with NIRS

**Fig. 1:**
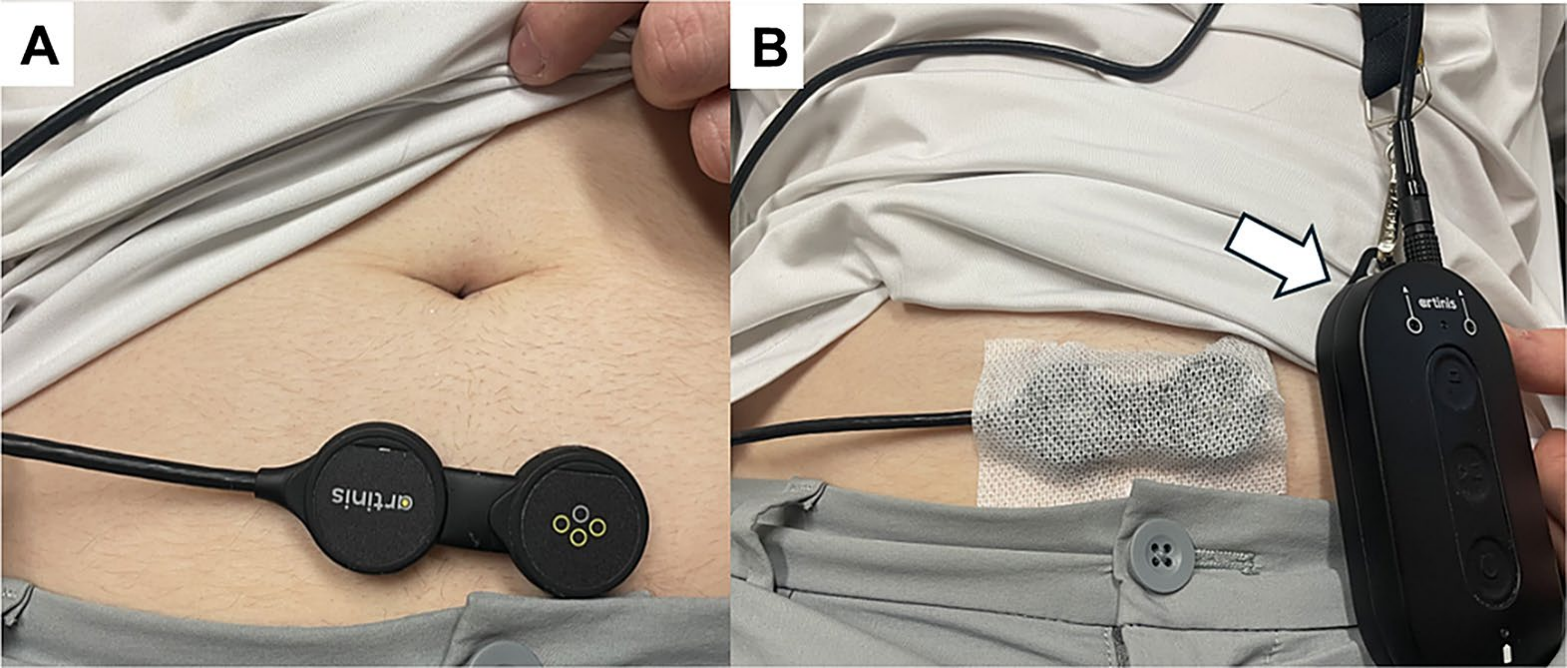
(**A**) Continuous O2Hb concentration was measured with the Portalite NIRS system, placed over the lower abdominal wall and covered with paper tape (**B**) to fix its position. The device contains an inertial measurement unit (IMU) or accelerometer to detect motion in three axes of direction and signals are recorded through a Bluetooth unit (arrow) placed near the device

While NIRS research has been promising in functional urology, inconsistent results and small single-institutional studies with small patient numbers have limited its scalability and widespread adoption for urologists and urogynecologists. The most notable issue that limits reproducibility is the presence of motion artifacts [30]. Previous studies excluded participants due to motion contamination, leading critics to dismiss the imaging technology as unreliable as a routine diagnostic tool [19]. However, substantial work has been performed to address motion artifact, making “modern” NIRS much more reliable for use in typical, ambulatory patients. Specifically, new NIRS devices include accelerometers called inertial measurement units (IMUs), which enable accurate detection and quantitation of motion in multiple spatial axes (Fig. 1). Ghatas et al. developed a method utilizing the NIRS IMU to identify and splice out areas of motion during NIRS data acquisition which can be used during natural filling studies looking at neuroexcitation in the cerebral cortex [31]. The group has now applied this technology to both the brain and bladder during UDS and natural filling and has demonstrated clear differences in NIRS patterns during bladder filling between controls and patients with OAB (Fig. 1). The team is also studying unique NIRS patterns associated with specific movements such as head tilts and waist bending.

**Fig. 2:**
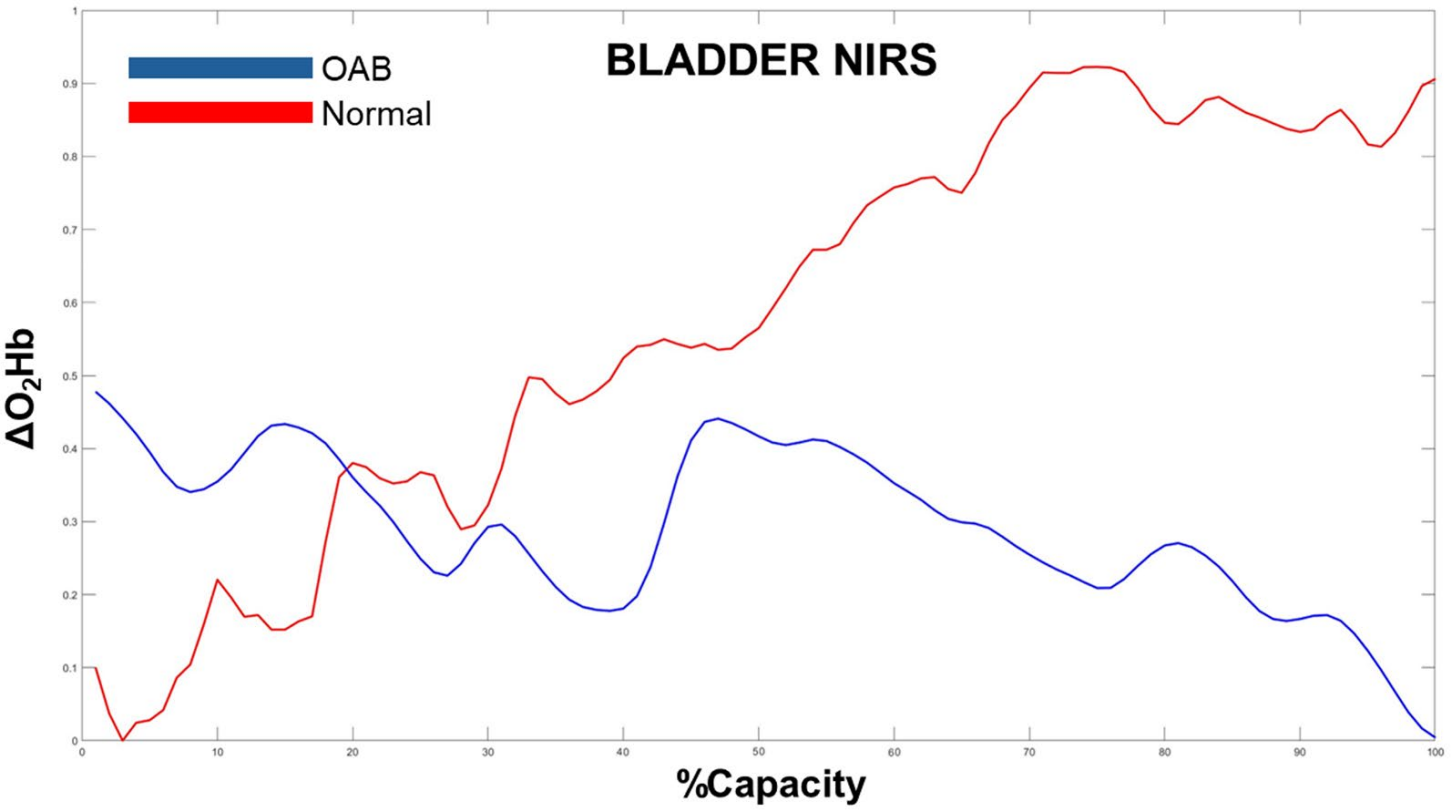
Example bladder NIRS signals showing change in oxyhemoglobin (AO2Hb, measured from baseline) for a normal female control (RED) and a female with OAB (BLUE). The x-axis represents percent capacity (bladder fullness). Our early studies demonstrate that normal individuals exhibit an increasing pattern in bladder NIRS, whereas those with OAB tend to show a flat or decreasing trend (unpublished)

In addition, wearability of NIRS devices has been a limiting factor. LED-based systems, though wireless, often have relatively large sizes and rigid designs, limiting their ability to be easily worn during activities of daily living. However, like other technology, modern NIRS devices are utilizing lightweight, flexible, wireless devices (Fig. 1), making wearability issues a concern of the past [14].

Finally, a major limitation of bladder NIRS is that current technology does not provide any information about bladder volume, an important component of UDS assessment of all forms of LUTD. However, there is ongoing work to address this issue [15]. Bladder volume monitoring has been conducted using NIRS by including a light source with a wavelength of 975 nm (the absorption peak of water). For example, Molavi et al. observed differences in light absorption between an empty and a full bladder [32]. From this finding, Fong et al. developed a machine learning algorithm that could predict bladder fullness using NIRS parameters. They were able to confirm a statistical difference between an empty bladder and a full bladder in a healthy participant [33]. Furthermore, ultrasound techniques, including wearable ultrasound technologies [34, 35], could also be used to non-invasively quantify bladder volume during NIRS assessment of bladder hemodynamics.

## NIRS Summary

NIRS technology offers the potential of a noninvasive device that could supplement or even replace traditional UDS, eliminating the risks, discomfort, and invasivity. Investigations now indicate that NIRS can serve as a new imaging modality for monitoring of the bladder and diagnosing LUTDs. Furthermore, many of the limitations of early NIRS devices (reproducibility, motion artifact, volume detection, wearability) have been addressed or improved with the current generation of NIRS technology. Still, with more research, NIRS represents a highly innovative technology on the horizon for the non-invasive diagnosis and evaluation of all forms of LUTD.

### Ultrasound

Using ultrasound imaging during traditional UDS can improve diagnosis of LUTD. This review of ultrasound imaging for improved UDS diagnostics focuses on ultrasound measurement of bladder volume, wall thickness, shape, vibrometry, elastography, and micromotion.

## Bladder Volume

In the push to develop non-invasive testing to substitute UDS, there is a need to identify real-time methods for measuring accurate bladder filling volumes during testing. One of the major advantages of UDS, is that the rate at which filling occurs is controlled by the urodynamicist, and the estimated bladder volume at any given point is known. Therefore, when an event such as first sensation or urge is felt, the volume can be easily recorded. Simple bladder scanning devices use ultrasound to assess bladder volumes; however, these widely available tools do not provide additional insight into anatomic and functional causes of LUTD. Vinod et al. conducted a study comparing a 2D bladder scanning device and 3D ultrasound to determine which device provides the most accurate estimate of bladder volumes during natural hydration studies. This study found that both modalities significantly underestimated true voided volumes. However, the group proposed the use of correction factors for both imaging modalities, which may improve measurement accuracy [36]. Similarly, another group found that bladder shape affects the accuracy of bladder scan volumes. The group proposed a correction coefficient based on bladder shape [37]. More recently, machine learning techniques have been used to improve the accuracy of ultrasound bladder volume quantification [34, 38, 39] and wearable ultrasound technologies for quantifying bladder volume have been developed [34, 35, 38]. Peng et al., used supervised learning to develop an algorithm to predict 3D bladder volume from 2D ultrasound images [39], and Cho et al., and Lee et al., used deep learning to estimate bladder volume using data from wearable ultrasound devices [34, 38].

Researchers have started to implement natural filling hydration protocols to replicate physiological filling. Sheen and colleagues, using ultrasound, performed a natural filling oral hydration study in healthy participants to validate a reproducible bladder filling protocol. In this study, bladder scan was used every 5 min while filling after an oral hydration period during two complete fill-void cycles. This study found that the initial fill cycle was a diuresis ramp-up phase with an accelerating rate of filling and an average fill rate of ~ 7 ml/min. Maximum diuresis was then achieved during the second fill at ~ 15 ml/min. These results were reproducible over multiple weekly visits [40]. Oral hydration protocols, along with wearable ultrasound real-time estimation of bladder volumes during filing [34, 35], present a potential method for non-invasive UDS with further refinement needed for accurate volume estimation.

## Bladder Wall Thickness

Bladder wall thickness (BWT) and bladder weight (BW) have previously been studied as possible measures of detrusor function. Ultrasound guided measurement of BW is found by subtracting the volume of the bladder lumen from the total bladder volume, assuming a spherical bladder shape [41]. BWT is determined by computing the average anterior wall thickness for at least three locations; however, this has previously been shown to vary during the early filling phase and stabilize once 50% bladder capacity has been achieved [42–44]. In more recent studies, BW and BWT show efficacy as a non-invasive method for diagnosis of BOO in men with LUTS. A 2023 metanalysis conducted by Chen et al. comparing bladder ultrasound metrics of more than 1800 men across 16 different studies found that BWT and BW diagnosed BOO with high sensitivity for both metrics. They found BWT had a sensitivity of 68% and a specificity of 91%, and BW had a sensitivity of 88% and a specificity of 81%. These results suggest that BWT and BW are both potentially useful in clinical application for non-invasive diagnosis of BOO. Additionally, BWT is positively correlated with symptom scores and has been shown to predict response to medical or surgical treatment of LUTS/obstruction [45]. Generally, the use of ultrasound in BOO and BPH has been established as an accurate and useful tool for clinical decision making in this setting. With further standardization, ultrasound guided measurement of BWT and BW may lead to improved diagnostic algorithms for BPH.

Furthermore, multiple investigators have studied BWT as a tool for identifying both DO and DU. Latthe et al. found that in women with OAB, BWT was not as good as UDS for the diagnosis of DO. In this study, women with OAB underwent transvaginal ultrasound to estimate BWT and UDS to identify which modality had a better rate of detection. BWT measured by transvaginal ultrasound had a sensitivity of 43% ultimately demonstrating little utility of BWT in the context of DO. In contrast, ultrasound may be more useful in the diagnosis of DU. In a study of 143 men with LUTS ultrasound BWT was measured at bladder volumes greater than 250 ml along with UDS. The study found that all men with BWT of less than or equal to 1.23 mm and bladder capacity greater than 445 ml had detrusor underactivity. BWT alone showed 87% accuracy in detecting DU [46]. De nunzio et al. expanded on this work by presenting a nomogram for the prediction of DU in men with LUTS. Furthermore, a multivariable logistic age-adjusted regression model found BWT and Qmax to be significant predictors of DU, again demonstrating the clinical applicability of BWT when treating individuals with significant LUTS [47].

### Bladder Shape

While the bladder is often modeled experimentally as spherical in shape, it is known that the shape of the bladder varies on a person-to-person basis. Factors such as pregnancy, severe adhesive bowel disease, and even severe constipation in children can alter the shape of the bladder leading to symptoms such as urgency and frequency [48]. In a study of women, including healthy volunteers and OAB participants, researchers found that transabdominal ultrasound in the transverse plane provided the most reliable imaging to characterize bladder shape. The group also found an increase in ellipsoid shapes in individuals with OAB, and that bladder shape changed during a bladder contraction [49]. However, another group used 3D ultrasound in a prospective study that found that bladder shape irregularities were more commonly identified using the sagittal imaging plane, and shape irregularities were more frequently identified in participants with OAB [50]. A later study, demonstrated that 3D ultrasound combined with an oral hydration protocol in healthy volunteers produced repeatable results measuring bladder shape across multiple fills and study visits [51].

Further studies have also shown that bladder shapes change throughout the filling process. In a 2020 study, researchers demonstrated in healthy female volunteers and women with OAB that changes bladder height are more pronounced than changes in different planes. In this study, bladder height-to-width ratios were measured in multiple imaging planes using transabdominal 3D ultrasound [52]. Height-to-width ratios were compared across participants at 20% capacity (low volumes) and 100 capacity (high volumes) to calculate strain. The group concluded that bladder filling mainly occurs in a vertical place. There was a key difference between OAB and healthy participants in that the OAB cohort were more likely to have less spherical bladders. These findings suggest that shape may play a role in the pathophysiology of OAB and proposed a potential shape mediated OAB phenotype.

**Fig. 3:**
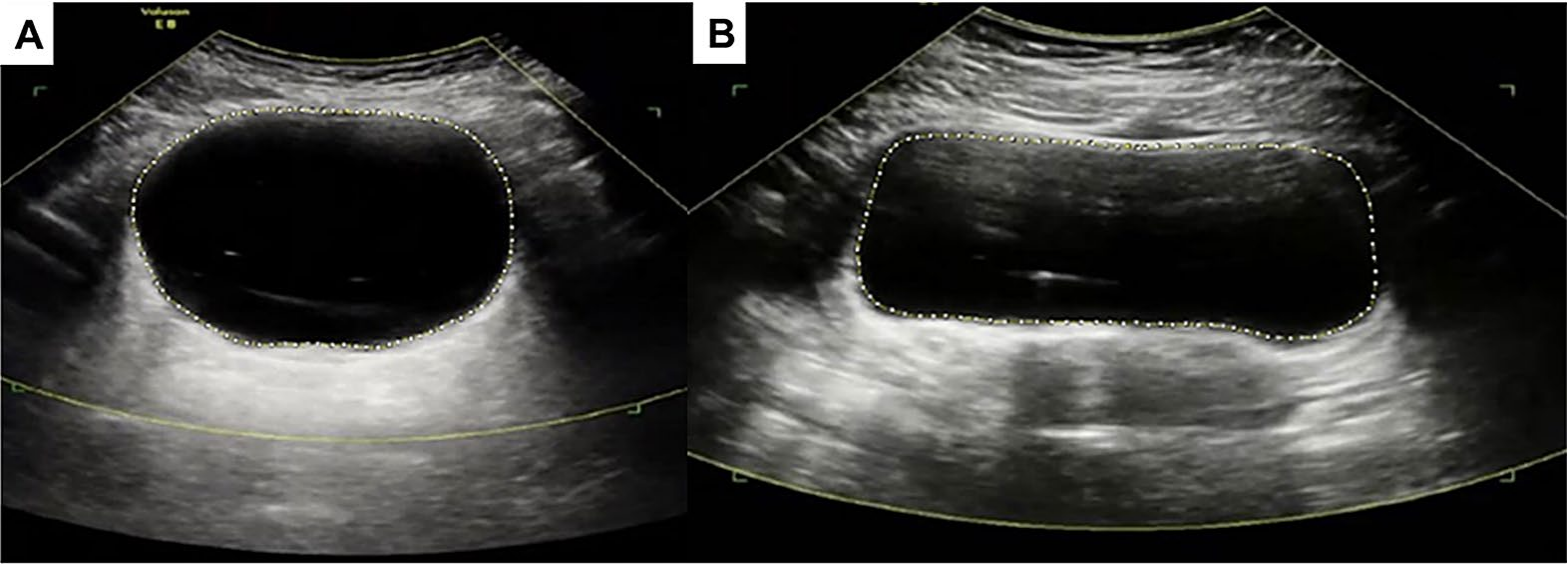
Ultrasound images with the bladder wall traced in ImageJ for (**A**) an individual with detrusor overactivity and circularity of 0.923 and (**B**) an individual without detrusor overactivity and circularity of 0.817

In addition, investigators have looked at bladder sphericity as an ultrasound-derived measure of DO [49]. They hypothesized that a bladder that is actively contracting will assume a more spherical shape. In this study, the investigators manually inscribed an ellipse within the lumen of the acquired transverse bladder image and a second ellipse around the entire bladder and defined a sphericity index based on the inner to outer ellipse ratio. Using this metric, they found that significant sphericity differences between individuals with and without DO. Likewise, recent work from the authors of this review (unpublished), shows similar findings using commercially available software to measure bladder circularity (Fig. 3).

### Elastography and Vibrometry

One of the gold standards of traditional UDS is measuring bladder compliance using intravesical pressures. Ultrasound bladder vibrometry presents a non-invasive alternative to this method. Researchers have used ultrasound to excite Lamb waves pulsation to measure the flexibility of the bladder wall [53]. In this study, the ultrasound emitted a pulsation and cross-spectral analysis was used to estimate the wave velocity, which was directly correlated to the elastic properties of the bladder. Building on this study, vibrometry was used to identify DO. In a study of participants with neurogenic bladder, researchers correlated bladder vibrometry measurements with DO identified during UDS to create a DO index [54].

Similar to vibrometry, elastography can be used to measure tissue stiffness. In this method, ultrasound sheer waves measure tissue stiffness in response to applied stress on the bladder wall [48]. This method was validated in a study with 23 pediatric patients with known bladder dysfunction. Researchers found that ultrasound sheer wave elastography measurements correlated strongly with bladder storage pressure, and that sheer wave speed measurements varied with bladder compliance. In a similar 2020 study of adults with suspected neurogenic bladder, elastography was shown to detect neurogenic bladder with high specificity in comparison to concurrent UDS studies. The study found that in patients with urodynamically proven DO and poor bladder compliance as markers of neurogenic bladder, sheer wave elastography was increased in 80% of the identified neurogenic patients [55]. Combined, these studies show that elastography can identify DO and neurogenic bladder dysfunction.

### Micromotion

**Fig. 4:**
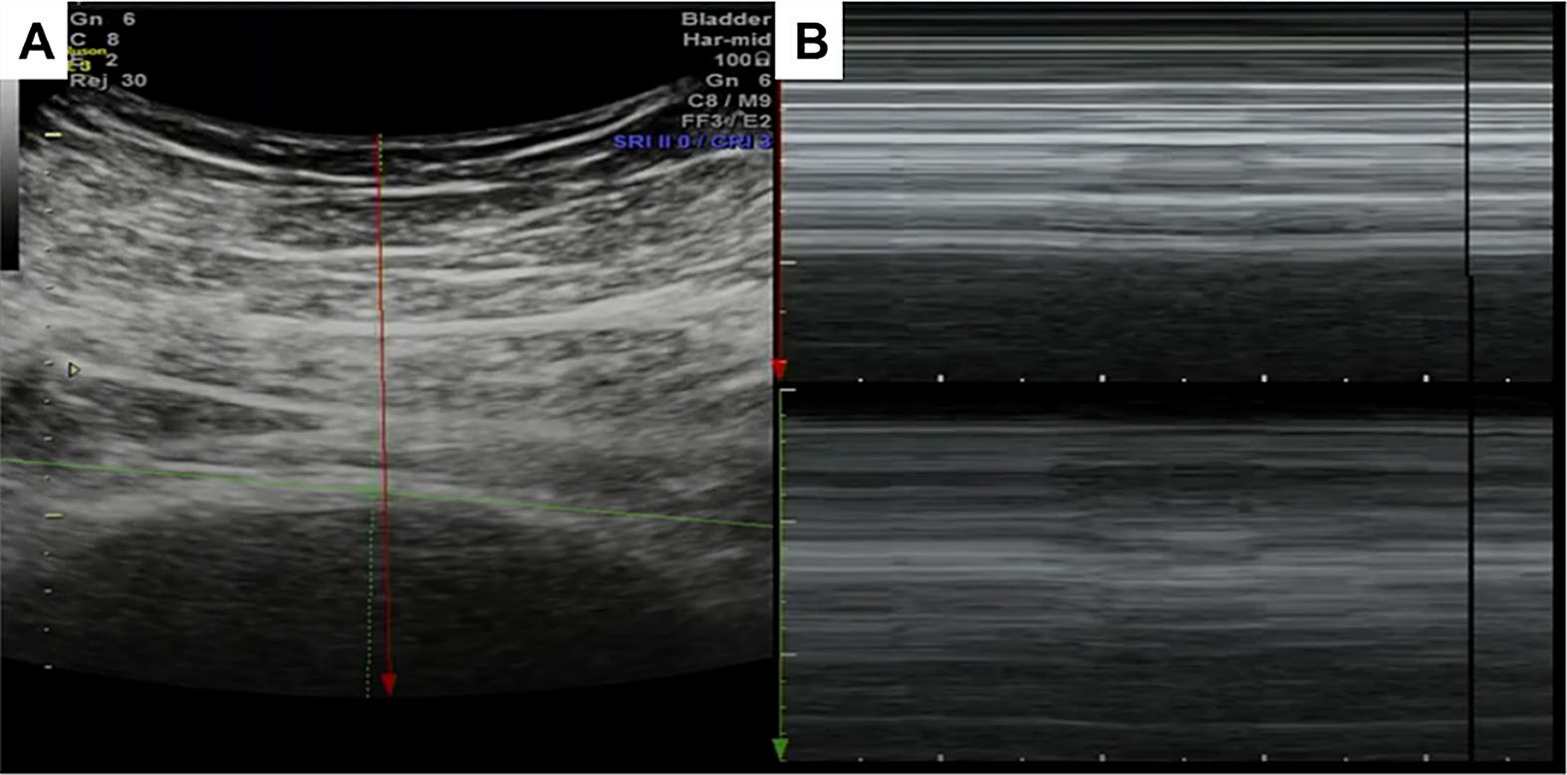
Static images of M mode bladder ultrasound acquired during urodynamic testing during filling. (**A**) The user manually identifies the outer and luminal bladder wall (green and red lines) and (**B**) a 1-dimensional bladder wall slice is displayed over time. A post-hoc texture tracking algorithm then records wall thickness over time as a measure of micromotion

Micromotion of the bladder wall due to localized contractions produces pressure changes associated with phasic DO during filling [56, 57]. Ultrasound has been used in previous studies as a non-invasive method to detect bladder wall micromotion. Using M-mode ultrasound, Nagle et al. demonstrated that micromotion correlated strongly with involuntary detrusor contractions [58]. The group used M-mode ultrasound to track the inner and outer luminal edges of the bladder wall over time. These measurements were used to calculate micromotion defined as a change in wall thickness of at least 0.1 mm. This study provides groundwork for future validation of ultrasound as a non-invasive means for detecting micromotion corresponding to DO [58]. An example of the technique is showing in Fig. 4.

### Dynamic MRI for LUT Biomechanics

fMRI studies have demonstrated the importance of brain processing in the control of micturition and in the potential pathophysiology of multiple forms of LUTD. However, the use of MRI to directly evaluate bladder function and LUT biomechanics is a more recent development [59]. Initial efforts to non-invasively assess the LUT used 2D acquisitions. These images were centered in the bladder neck or urethra with the goal of evaluating anatomical differences while the subjects were performing a valsalva maneuver, using a combination of static images and performing a timed analysis. Real-time dynamic MRI in the LUT began with the study of bladder prolapse, initially using T2 ultra turbo and turbo spin echo [60–62] sequences, later advancing to steady-state free presession (true-FISP) sequence [63, 64]. Urethral mobility has been imaged and evaluated using the HASTE sequence [65]. Ureter peristalsis has been captured and studied using oblique planes passing through the ureters and using the TWIST (Time-resolved angiography with stochastic trajectories) sequence [66].

**Fig. 5:**
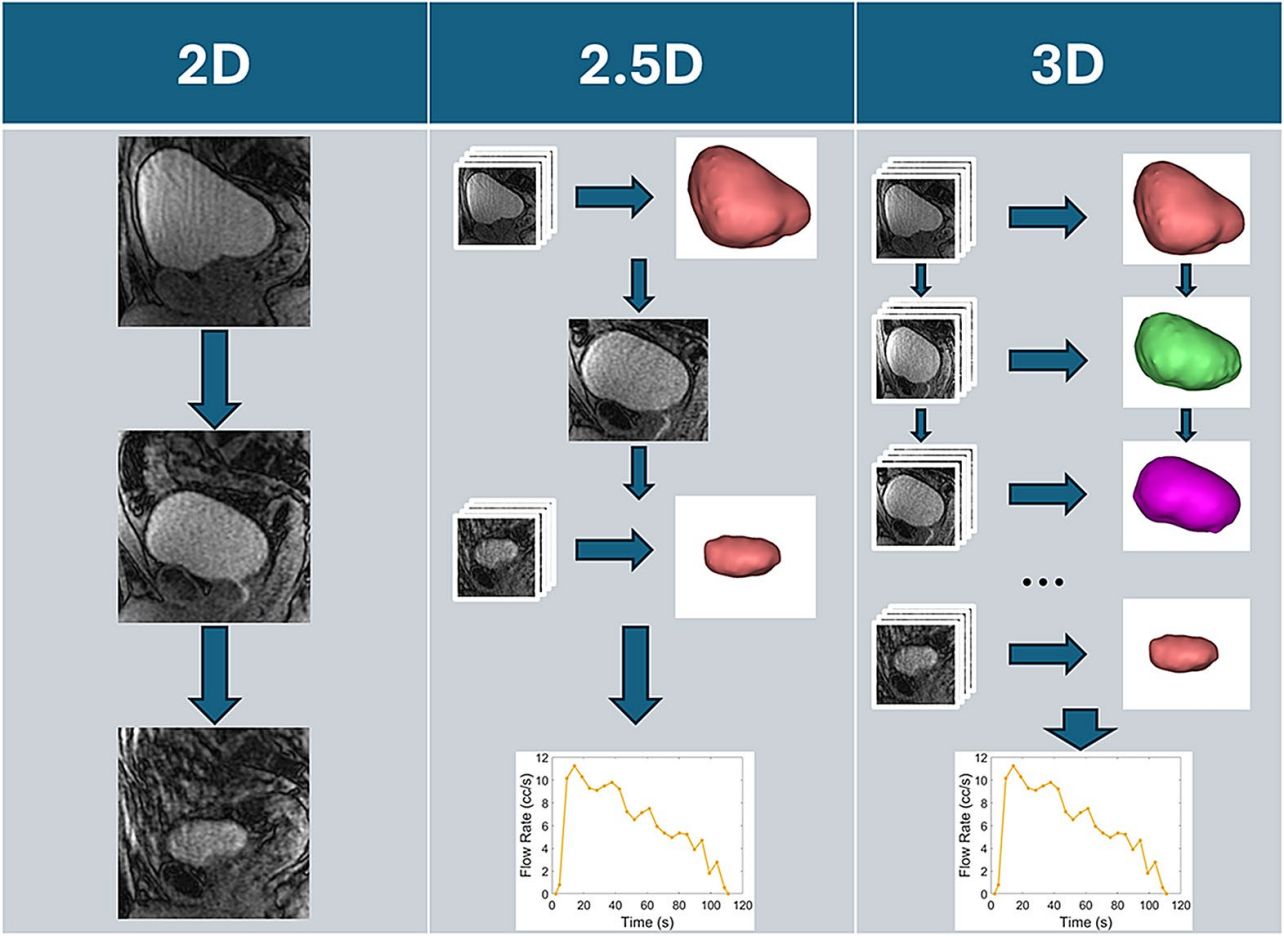
Visual representation of the evolution of dynamic MRI for the evaluation of the LUT, starting with 2D, following with 2.5D and ending with 3D. Initial methodologies use extended acquisition times or real-time acquisition times to quantify in-plane anatomical changes in the LUT. 2.5D methodologies combine volumetric (3D) acquisitions with extended acquisition times with real-time 2D acquisitions to interpolate volumetric changes during diverse processes in the LUT. 3D methodologies use contrast-enhanced volumetric acquisitions with short acquisition times to capture dynamic processes in the LUT such as bladder voiding and filling

More recently, studies have shown the combination of pre and post voiding static 3D Fast Spin Echo (FSE) T2 weighted acquisitions and dynamic sagittal 2D SGRE (Spoiled Gradient Echo) acquisitions combined with computational fluid dynamics (CFD) to evaluate bladder voiding mechanics [67]. From these efforts, contrast-enhanced 3D dynamic sequences based on 3D Differential Subsampling with Cartesian Ordering (DISCO) have been developed to capture the entirety of the LUT every couple of seconds [68, 69]. These methods have also been coupled with CFD [59] to create a comprehensive evaluation of the LUT using dynamic MRI (Fig. 5).

These advances in both MRI technology and acquisition technique have paved the way for future studies which can produce high resolution 3D bladder images coupled with functional data for the evaluation of LUTD.

## Conclusion

Traditional multi-channel UDS remains the gold standard for the assessment of LUTD. However, the limitations of UDS technology including poor reproducibility and invasiveness have resulted in development and study of novel imaging techniques which can supplement or even replace UDS. New advances have been achieved in multiple bladder imaging modalities including bladder NIRS, ultrasound, and dynamic MRI. These developing techniques have the advantage of being non-invasive and, in many cases, can provide high-resolution anatomic images alongside functional bladder data. While these techniques are not yet ready for widespread adaptation into clinical practice, it is a certainty that the future of functional bladder assessments will include novel imaging modalities. Keep a close eye on the imaging horizon!

### Key References

* = Significant.

** = Very Significant.

**   Ghatas MP, Burkett LS, Grob G, et al. A stepwise approach for functional near infrared spectroscopy measurement during natural bladder filling. *Transl Androl Urol*. Oct 31 2023;12 [10]:1477–1486. 10.21037/tau-23-275.
  - This study details new technology and step-by-step methodology to overcome motion artifact one of the key issues limiting more widespread adoption of NIRS into clnical practice.
**   De Nunzio C, Lombardo R, Cicione A, et al. The role of bladder wall thickness in the evaluation of detrusor underactivity: Development of a clinical nomogram. *Neurourology and urodynamics*. Apr 2020;39 [4]:1115–1123. 10.1002/nau.24327.
  - This paper describes a clinical nomogram, including direct ultrasound measurements of bladder wall thickness, to provide a novel tool for the objective diagnosis of detrusor underactivity.
**   Shahid L, Gonzalez-Pereira JP, Johnson C, Bushman W, Roldan-Alzate A. Computational fluid dynamics of bladder voiding using 3D dynamic MRI. Int J Numer Method Biomed Eng. 2024;40 [9]:e3850.
  - This feasiblity study describes the application of magnetic resonance imaging (MRI)-based computational fluid dynamics, developed in the study of cardiovascular function, to the study bladder voiding.
*   Koven A, Herschorn S. NIRS: Past, Present, and Future in Functional Urology. Curr Bladder Dysfunct Rep. 2022;17 [4]:241–249. 10.1007/s11884-022-00665-4.
  - This paper reviews the history of NIRS in urology and how the technology has advanced over time.
*   Glass Clark S, Nagle AS, Bernardo R, et al. Use of Ultrasound Urodynamics to Identify Differences in Bladder Shape Between Individuals With and Without Overactive Bladder. Female Pelvic Med Reconstr Surg. Oct 2020;26 [10]:635–639.
  - This study demonstrates how bladder shape, measured using ultrasound during urodynamics, can be used to potentially identify a shape-mediated sub-type of overactive bladder.
*   Macnab AJ, Stothers L. Near-infrared spectroscopy: validation of bladder-outlet obstruction assessment using non-invasive parameters. Can J Urol. 2008;15 [5]:4241-8.
  - This study describes the use of a simple, lightweight, device that can accurately (> 85%) predict bladder out let oubstruction using the combined parameters of NIRS, post-void residual, and maxium uroflow rate.
*   Lee K, Lee MH, Kang D, Kim S, Chang JH, Oh SJ, Hwang JY. Intelligent Bladder Volume Monitoring for Wearable Ultrasound Devices: Enhancing Accuracy Through Deep Learning-Based Coarse-to-Fine Shape Estimation. IEEE Trans Ultrason Ferroelectr Freq Control. 2024;71 [7]:775 − 85.
  - A key need in the development of urodynamic imaging techniques is the ability to accurate estimate volume, non-invasively. This paper describes the use of artifical intelligence to optimize non-invasive bladder volume estimation with wearable ultrasound devices.

## Data Availability

No datasets were generated or analysed during the current study.
